# Hydrogen Peroxide Sensing Based on Inner Surfaces Modification of Solid-State Nanopore

**DOI:** 10.1186/s11671-017-2190-x

**Published:** 2017-06-20

**Authors:** Libo Zhu, Dejian Gu, Quanjun Liu

**Affiliations:** State Key Laboratory of Bioelectronics, School of Biological Science and Medical Engineering, Southeast University, No. 2, Sipailou, Nanjing, 210096 People’s Republic of China

**Keywords:** Sensor, Nanopore, Hydrogen Peroxide, Horseradish Peroxidase (HRP)

## Abstract

There are many techniques for the detection of molecules. But detection of molecules through solid-state nanopore in a solution is one of the promising, high-throughput, and low-cost technology used these days. In the present investigation, a solid-state nanopore platform was fabricated for the detection of hydrogen peroxide (H_2_O_2_), which is not only a label free product but also a significant participant in the redox reaction. We have successfully fabricated silicon nitride (Si_3_N_4_) nanopores with diameters of ~50 nm by using a focused Ga ion beam, the inner surface of the nanopore has been modified with horseradish peroxidase (HRP) by employing carbodiimide coupling chemistry. The immobilized HRP enzymes have ability to induce redox reactions in a single nanopore channel. Moreover, a real-time single aggregated ABTS^•+^ molecular translocation events were monitored and investigated. The designed solid-state nanopore biosensor is reversible and can be applied to detect H_2_O_2_ multiple times.

**Electronic supplementary material:**

The online version of this article (doi:10.1186/s11671-017-2190-x) contains supplementary material, which is available to authorized users.

## Background

Nanopore detection technology originates from Coulter counter [1] and cell ion channel [2]. Nanopore detects charged molecules present in a solution passing through it. The appearance of the molecules in nanopore can change the conductance of the pore apparently, consequently a change in the current signal. The change in the current provides the information about the sizes and concentration of the molecules inside the pore, to reveal the dynamics process of the molecules translocation behaviours [3]. Some nanoscale objects can be detected using a nanopore, such as nanoparticles [4–6], viruses [7–9], protein molecules [10–13] and DNA sequences [14–17]. Nanopores are of two types. Biology nanopore and the solid-state nanopore. The biology nanopore has lower signal to noise ratio (SNR), and higher resolution. Small and unfolded proteins can be detected by using biology nanopores [18–23]. Solid-state nanopore is size adjustable and has higher stability. The solid-state nanopore is normally drilled on a film, this film divides the fluidic cell into two parts [24]. A biased voltage is applied across a thin membrane containing a nanopore, resulting in an ionic current from one cell to another [25]. Protein molecules including folded and unfolded structures are detected and analyzed by solid-state nanopore [26–29]. The interaction of proteins can also be detected using solid-state nanopore [30, 31]. Moreover, it has ability to detect protein kinetics [32, 33]. In order to solve the limits on the detection range, chemically modified solid-state nanopores have been applied extensively [34–39], chemically modified solid-state nanopores have been applied to detect single-stranded DNA [40] and proteins [41].

A lot of quantitative methods have already been applied for the detection of H_2_O_2_, most of them are based on spectrometry [42–45], chemoluminescence [46–49], amperometry [50–53] and electrochemistry [54–57]. The conventional spectrometric and chemoluminescence methods are commonly time-consuming and costly. The solid-state nanopore sensor has low consumption and simple structure, and can be used to detect small molecules.

Here, we present a type solid-state nanopore that was modified with horseradish peroxidase (HRP). The HRPs were immobilized on the inner surface of solid-state nanopore, the immobilized HRPs remained active in redox reaction that occurred inside a single nanopore channel in the presence of H_2_O_2_ [58]. The ABTS^•+^ produced in redox reaction would aggregate, then the aggregated ABTS^•+^ passed through nanopore. The translocation events can be detected. For the hydrogen peroxide detection, the structure of solid-state is simple, and it can detect the aggregated ABTS^•+^ by using low reagent consumption. This horseradish peroxidase (HRP) enzymes modification solid-state nanopore can accomplish the hydrogen peroxide (H_2_O_2_) sensing indirectly, through the aggregated ABTS^•+^ detection. It has instructive significance for single molecule detection and molecules assembly inner solid-state nanopore.

## Methods

### Chemicals and Materials

The Horseradish Peroxidase (HRP) molecule (1mg mL^-1^, Enzyme Commission No.1.11.1.7, 44 kDa) was purchased from Xiya Reagent (Chengdu, China). The sample (HRP) was dissolved in 0.02 μm filtered 0.1 M PBS, stored at 4 °C, and employed within two days of preparation. Potassium chloride (KCl), N-(3-dimethylaminopropyl)-N’-ethylcarbodiimide (EDC), N-hydroxysuccinimide (NHS) and 2,2’-Azino-bis (3-ethylbenzothiazoline-6-sulfonic acid) ((ABTS), 98%) were purchased from DiBo chemical technology co., LTD (Shanghai, China). Hydrogen peroxide (H_2_O_2_, 30%) was bought from Sinopharm Chemical Reagent Co., Ltd. (3-Aminopropyl)triethoxysilane (3-APTES) was purchased from Sigma-Aldrich (St. Louis, MO, USA). Experiments were conducted using untrapure water from a Milli-Q water purification system (resistivity of 18.2 MΩ/cm, 25 °C, Millipore Corporation, Billerica, MA, USA) and was filtered through 0.02 μm in a FEI Strata 201 FIB system (FEI Co., Hillsboro, OR, USA), a Zetasizer (Malvern Zetasizer Nano ZS), and an Axopatch 700B (Molecular Devices, Inc., Sunnyvale, CA, USA). The pictures of our used instruments were added to the supplementary material (see Additional file 1: Figure S1).

### Solid-State Nanopore Fabrication and Electrical Measurements

First, a thin membrane of Si_3_N_4_ (100 nm thickness) was deposited on a Si substrate having 300 μm thickness. Followed by photolithography (the open window size is 500 × 500 μm^2^). Then, the surface of the membrane was bombarded with Ga + ions using a FEI Strata 201 FIB system (FEI Co., Hillsboro, OR, USA) at an acceleration potential of 30 kV, while the current was measured as 1 pA. The milling time was 1.5 s under a spot mode. Finally, the solid-state nanopore chips were obtained and cleaned in fresh prepared piranha solution at 80 °C for 30 min, followed by rinsing with ultrapure water. After cleaning, the chip was assembled in a custom-built Teflon cell with two Viton o-rings to separate the two sides of chip, and forming two reservoirs to ensure the only path for ionic current through the nanopore. The pictures of our used apparatus were added to the supplementary material (see Additional file 1: Figure S2). Electrodes (Ag/AgCl) were connected to the fluidic cell and a patch clamp amplifier (Axopatch 700B, Molecular Devices, Inc., Sunnyvale, CA, USA) that made the ionic current measurable under constant voltages, with 100 kHz sampling rate for signals. The amplifier internal low-pass eight-pole Bessel filter was set at 10 kHz [3]. The whole instrument was placed in a double Faraday cage enclosure.

## Results and Discussion

### Immobilization of Nanopore with HRPs

The selected nanopore with a diameter of ~50nm was immersed in piranha solution at 80 °C for 30 min. After treating with piranha solution, the inner surface of nanopore was able to take silicon hydroxyl groups. Subsequently, the entire thin film was activated with (3-Aminopropyl)triethoxysilane (3-APTES). As a result of treating with 3-APTES, the amino (-NH_2_) groups were generated on the surface of film.

**Fig. 1:**
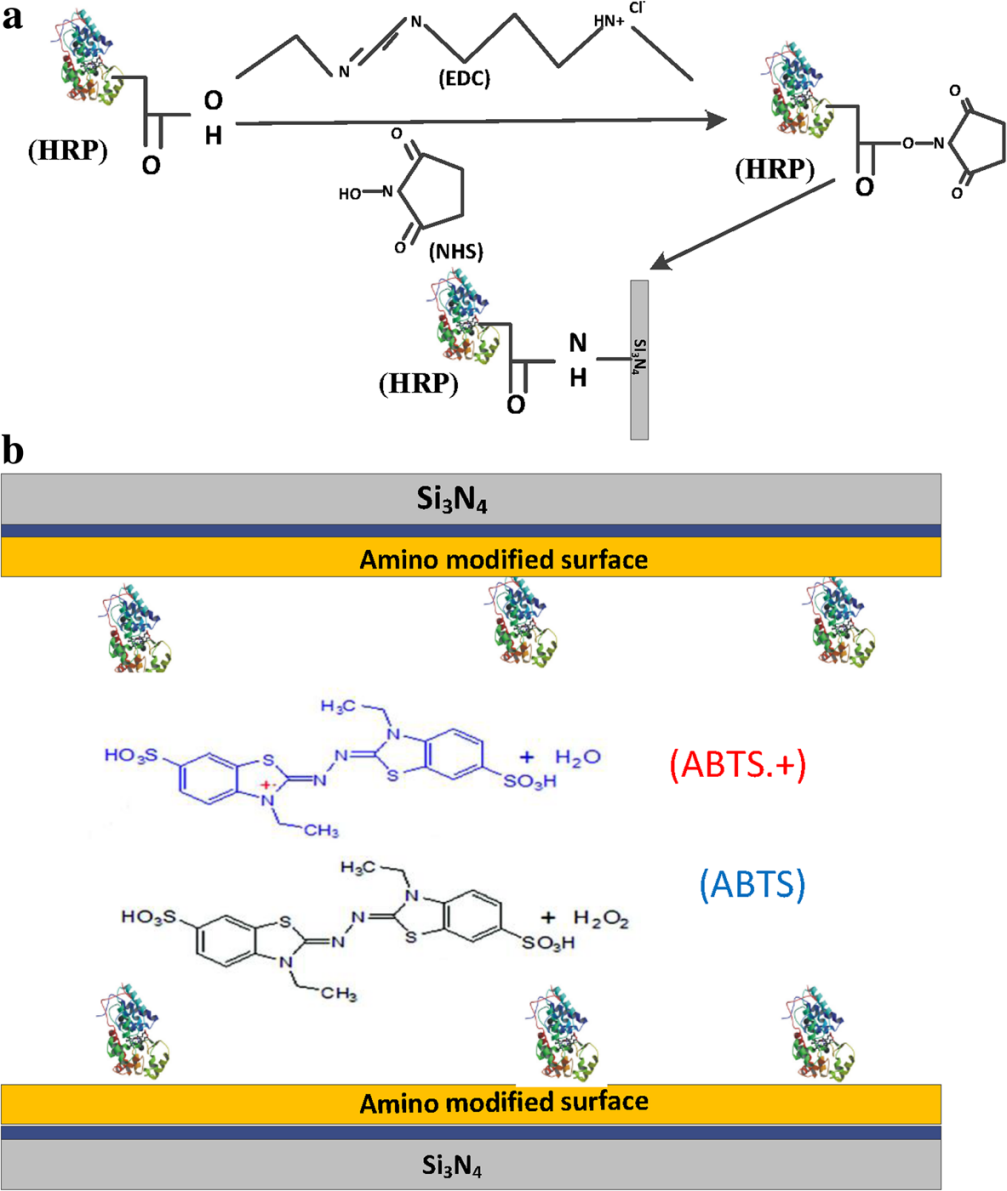
Performing modification process in a single solid-state nanopore channel. **a** Schematic representation of the covalent attachment of horseradish peroxidase (HRP) to a single nanopore channel via carbodiimide coupling chemistry. The carboxyl (-COOH) groups of HRP was activated by EDC solution, enabling the HRP to react with (-NH_2_) generated on the surface of a nanopore chip. **b** Scheme of the immobilized HRPs hydrogen peroxide sensor, the inner surface of sensor was modified with HRPs. When H_2_O_2_ and ABTS occurred, the ABTS^•+^ produced. The crystal structure of HRP was used with the permission from author [3]

After activation with (3-Aminopropyl)triethoxysilane (3-APTES), the nanopore chip was brought into 0.1 M PBS solution of N-(3-dimethylaminopropyl)-N’-ethylcarbodiimide (EDC) (10 mM) and N-hydroxysuccinimide (NHS) (20 mM). Thereafter, the nanopore chip was introduced to horseradish peroxidase (HRP) (10 ng/ml). According to previous research results of our group [3], with different salt concentration from 0.1 to 2 M KCl, pH 7.0, HRP did not aggregate. On account of the *pI* value of horseradish peroxidase being 4.3 ± 0.2, we also proved that the HRP did not aggregate in 0.1 M KCl pH 6.0 and pH 7.0. The EDC reagent activated the carboxyl (-COOH) groups of HRP into a highly reactive o-acylisourea intermediate. Furthermore, the intermediate was further converted into a more stable succinimidyl amine-reactive ester in the presence of NHS [58]. Resulting, in covalent coupling of the intermediate with the (-NH_2_) generated on the inner surfaces of nanopore to form stable amide bonds (Fig. 1).

**Fig. 2:**
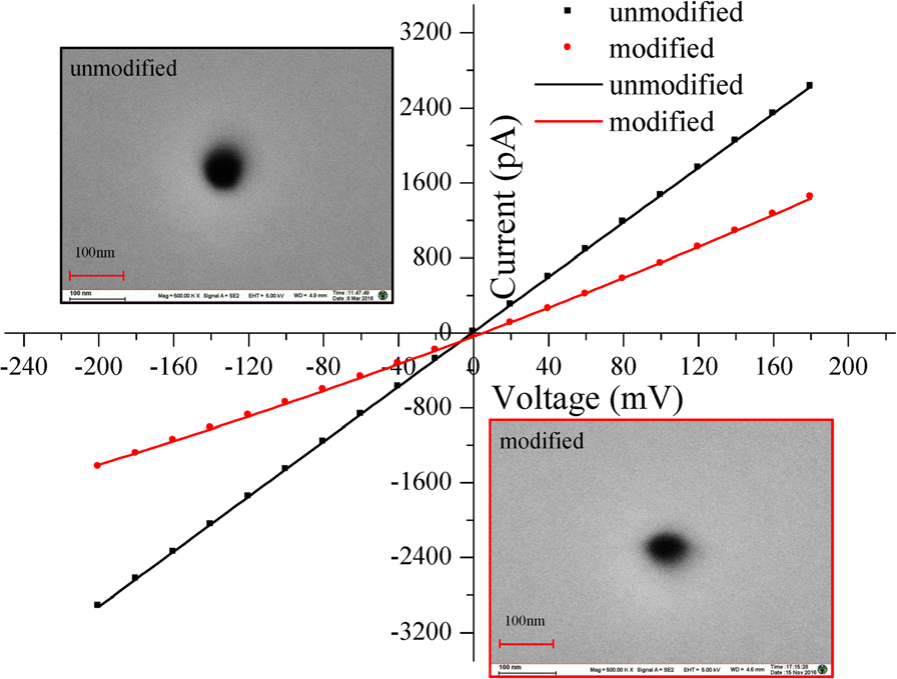
Performing a typical current-voltage (*I-V*) curves of the unmodified (original) and modified nanopore in 0.1 M KCl, buffered at pH 7.0 with 0.1 M PBS. The *black line* is the *I-V* curves of unmodified nanopore, and *red line* is the *I-V* curves of modified nanopore with HRPs. The *inserts* are the scanning electron microscopy (SEM) of a single nanopore (diameter of ~50 nm cis) and the nanopore sensor. 100 nm is scale

These processes lead us toward the immobilization of HRPs on the inner surface of a single nanopore. The realization of functionalization process was confirmed by measuring the current-voltage (*I-V*) of a single nanopore before and after modification (Fig. 2).

### Characterization of HRPs Modified Solid-State Nanopore

Here, the shape of a single Si_3_N_4_ nanopore channel is cylindrical. Figure 2 shows the typical current-voltage (*I-V*) curves of the unmodified (original) and modified nanopore in 0.1 M KCl, buffered at pH 7.0 with 0.1 M PBS. After modifying the inner surface of nanopore with HRP enzymes, the pore size became smaller.

According to Wanunu et al, by taking the conductance of external of nanopore into account, diameter of solid-state nanopore can be calculated by the following equation,

1 $$ d=\left(1+\sqrt{1+\frac{16\sigma l}{\pi G}}\right) G/2\sigma $$

Where, *d* and *l* are the diameter and length of pore,*G* is open pore conductance of nanopore, *σ* is the conductivity of ion solution.

Considering geometric effects, after the modification of solid-state nanopore with HRP enzymes, effective size can be calculated. The diameter of a single nanopore can be calculated based on the equation (1). Where, the value of conductance (*G*_*unmodified*_) is ~15 nS can be obtained from *I-V* curves of the unmodified solid-state nanopore. The conductivity (*σ*) of ion solution 0.1 M KCl (25 °C), buffered at pH 7.0 with 0.1 M PBS is ~1.28 S/m. Therefore, the diameter of unmodified nanopore is ~51 nm, it is similar to the measured diameter. Using the same method, the value obtained of conductance (*G*_*modified*_) is ~7.5 nS, and the diameter (~34 nm) of modified nanopore can be calculated. Reduction in diameter is possible due to the following two reasons, first is treating the inner surface of nanopore with (3-aminopropyl)triethoxysilane (3-APTES), allowing the surface of nanopore to take (-NH_2_) amino groups. The second reason is that the hydrodynamic diameter (*D*_*h*_) of HRP enzyme is ~8 nm [3], the immobilized HRPs could reduce the pore’s diameter. Here, the HRPs modified solid-state nanopore with diameter of ~34 nm is used as the hydrogen peroxide detection channel.

### The Principle of Redox Reaction

The redox reaction was conducted inside a single modified nanopore, and the following presented reaction process agrees well with the redox reaction proposed [58]. In presence of H_2_O_2_ (0.5 mM), HRP enzymes immobilized on the inner surface of nanopore was converted into compound 1 immediately. Then, the compound 1 accepted one electron from the reducing substrate molecule ABTS (1.5 mM) to generate compound 2. Subsequently, compound 2 was reduced back to the resting enzyme via one electron transfer from another substrate molecule ABTS.

The cationic products (ABTS^•+^) of the redox reactions were accumulated in single nanopore. The translocation of accumulated molecules from the nanopore channel would change the conductance (*G*), and thus the change of current (*ΔI*_*b*_) can be found.

$$ \mathrm{H}\mathrm{R}\mathrm{P}\left({\mathrm{Fe}}^{3+}\right)\mathrm{Porp} + {\mathrm{H}}_2{\mathrm{O}}_2\to \mathrm{H}\mathrm{R}\mathrm{P}\left({\mathrm{Fe}}^{4+}=\mathrm{O}\right){\mathrm{Porp}}^{\cdotp +}\left(\mathrm{Compound}\ 1\right) + {\mathrm{H}}_2\mathrm{O} $$

$$ \mathrm{H}\mathrm{R}\mathrm{P}\left({\mathrm{Fe}}^{4+}=\mathrm{O}\right){\mathrm{Porp}}^{\cdotp +} + \mathrm{ABTS}\to \mathrm{H}\mathrm{R}\mathrm{P}\left({\mathrm{Fe}}^{4+}=\mathrm{O}\right)\mathrm{Porp}\left(\mathrm{Compound}\ 2\right) + {\mathrm{ABTS}}^{\cdotp +} $$

$$ \mathrm{H}\mathrm{R}\mathrm{P}\left({\mathrm{Fe}}^{4+}=\mathrm{O}\right)\mathrm{Porp} + \mathrm{ABTS}\to \mathrm{H}\mathrm{R}\mathrm{P}\left({\mathrm{Fe}}^{3+}\right)\mathrm{Porp} + {\mathrm{ABTS}}^{\cdotp +} + {\mathrm{H}}_2\mathrm{O} $$

### Detection of Translocation Events

**Fig. 3:**
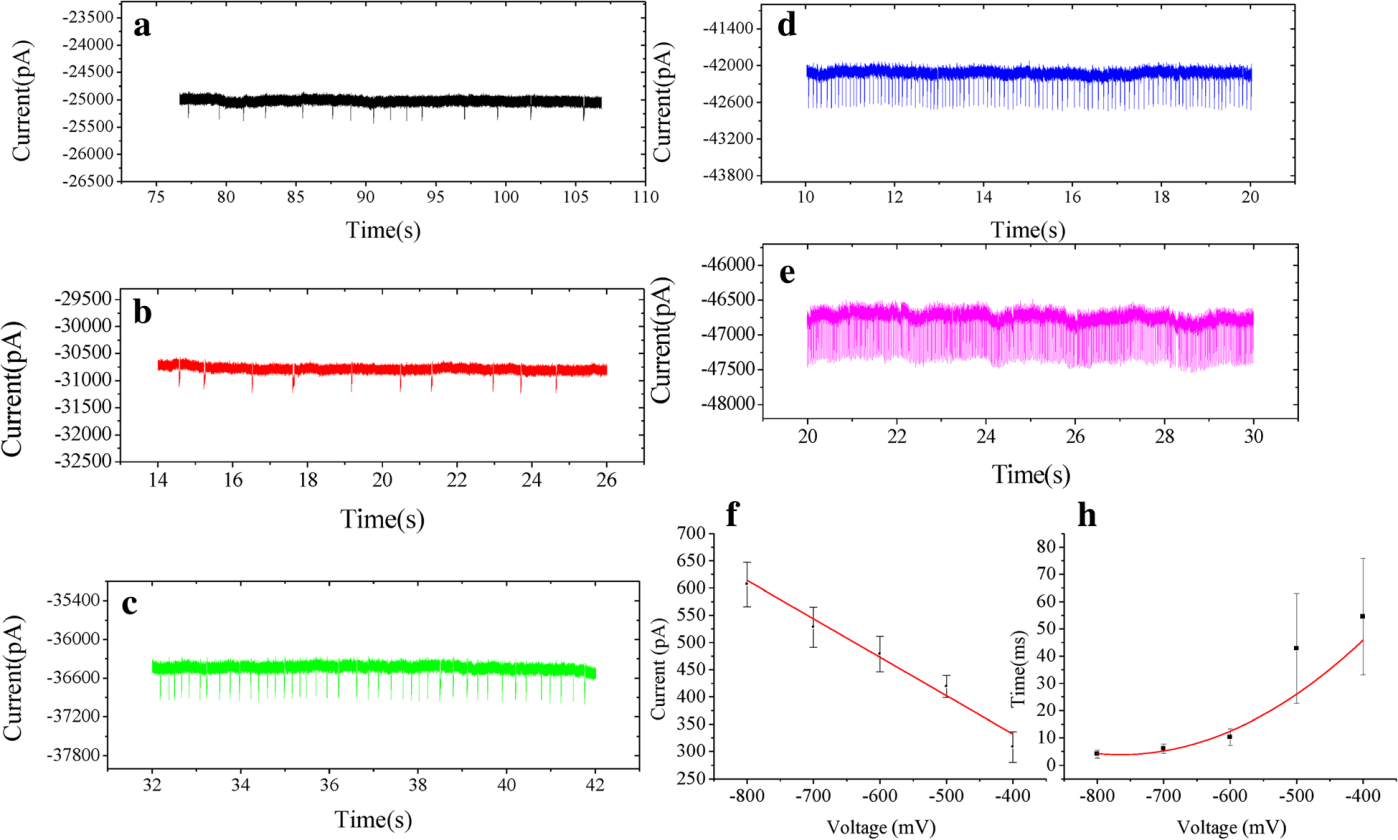
**a**–**e** Schematic representation of the translocation events at different voltages from −400 to −800 mV. The frequency of translocation events increased when the applied voltage increased from −400 mV to −800 mV. **f** The current amplitude linearly increases with the voltage. **h** An exponentially decaying function (*t*_*d*_ *~ e* ^*−v/v0*^) was employed to fit the dwell time dependent on the applied voltages

Experiments were performed using horseradish peroxidase (HRP) modified nanopores with modified pore diameters (~34 nm) in 0.1 M KCl, buffered at pH 7.0 with 0.1 M PBS. 2, 2'-Azinobis-(3-ethylbenzthiazoline-6-sulphonate (ABTS) (1.5 mM) and hydrogen peroxide (H_2_O_2_) (0.5 mM) were added to the trans compartment of the nanopore. After adding ABTS and H_2_O_2_, the experiments using biased voltages from −100 to −800 mV were performed, and they were sampled at 100 kHz. There were no translocation event until the voltage increased to −400 mV. Figure 3 shows representative ionic current traces of the translocation event at different voltages from −400 to −800 mV in 0.1 M KCl, 0.1 M PBS, pH 7.0. The experiments data of long duration translocation events of different voltages were added to the supplementary material (see Additional file 1: Figure S3).

The millisecond current blockade events were observed, conducted in 0.1 M KCl, 0.1 M PBS, pH 7.0. By adding the reagent H_2_O_2_ and ABTS into the transcompartment, HRP enzymes immobilized on the inner surface of single nanopore channel, and redox reaction occurred. Abundance of ABTS^•+^ molecules were produced, a single smaller molecule ABTS^•+^ may not be detected using our solid-state nanopore, due to the resolution of the system [3]. However, these molecules would aggregate after their production. Therefore, it is possible to detect the ABTS^•+^ molecules. Here, the negative voltages were held, and aggregated ABTS^•+^ molecules passed through the nanopores. There was an electrophoretic and eletroosmotic flow when negative voltage was applied. HRP was negatively charged in 0.1 M KCl, 0.1 M PBS, pH 7.0 [3], as a result, double electrical layer would be produced, and eletroosmosis would be towards negative electrode direction. Due to this reason, electrophoresis and eletroosmosis were towards to the same direction. The aggregated ABTS^•+^ in the single nanopore channel would transport through the solid-state nanopore, flow toward negative electrode direction.

### Statistical Analysis of Translocation Events

**Fig. 4:**
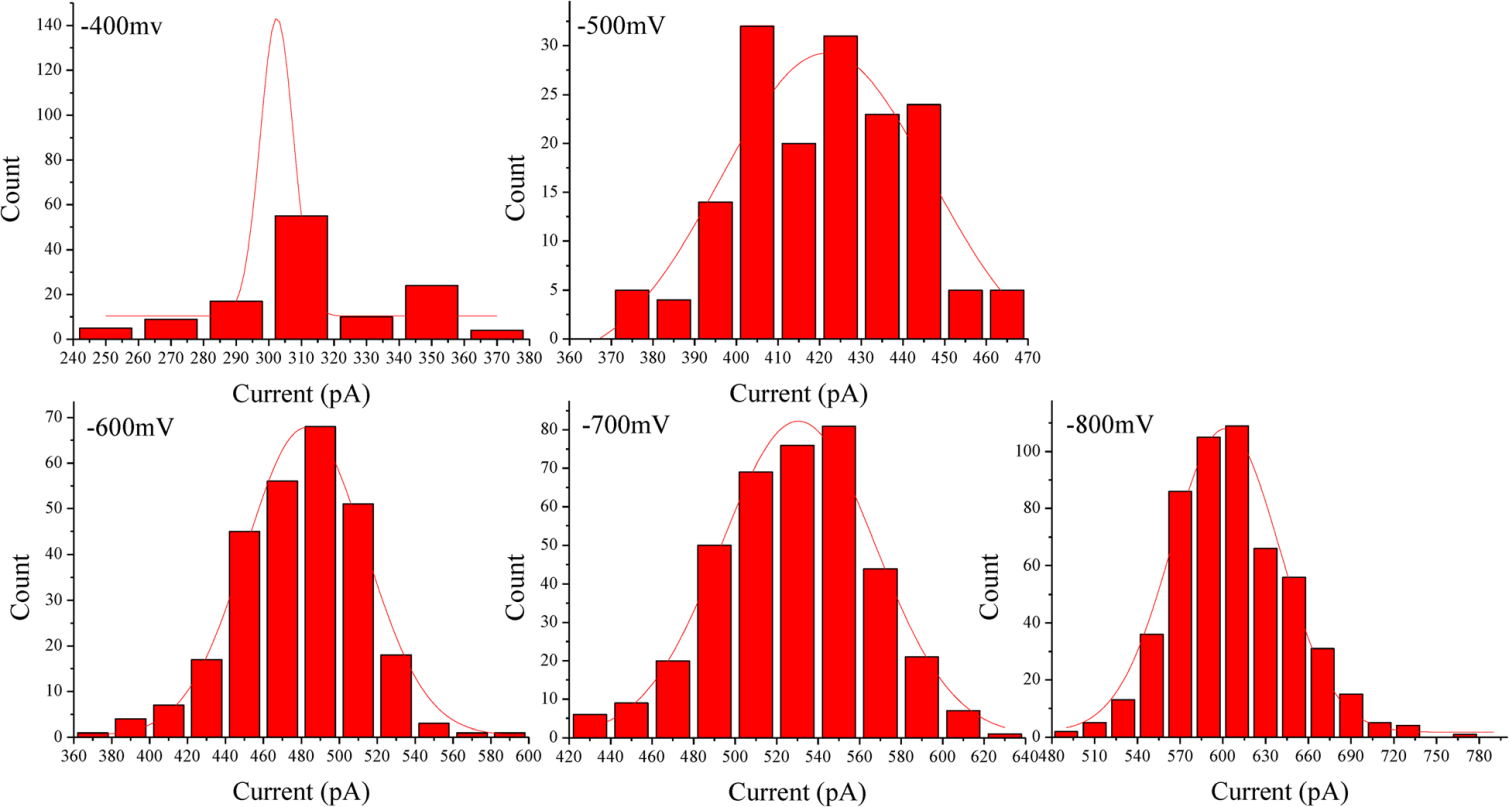
The histograms of the mean current amplitude of translocation events measured for aggregated ABTS^•+^ at different voltages (−400, −500, −600, −700, −800 mV) in 0.1 M KCl, 0.1 M PBS, pH 7.0. All histograms were fitted with Gaussian distribution

Since the biased voltage played a key role in translocation of aggregated ABTS^•+^, the influence of current blockades of aggregated ABTS^•+^ passing through the HRPs modified nanopore versus applied voltages was discussed. The occurrence frequency of translocation events was greatly improved with the increase in voltage (Fig. 3f). As the voltage increased, so does the amplitude of the current. However, the translocation events gradually disappeared when the biased voltage was kept below −300 mV, which suggested that aggregated ABTS^•+^ across HRPs modified nanopores needed a −300 mV threshold voltage. Figure 4 shows histograms of the mean current amplitude of translocation events measured for aggregated ABTS^•+^ at different voltages. Based on the fitting curves, the peak values of the current blockage (*ΔI*_*b*_) are 308.4 ± 27.795 pA, 419.1 ± 20.354 pA, 478.8 ± 32.857 pA, 528.1 ± 36.98 pA, 606.9 ± 40.916 pA at −400, −500, -600, −700, and −800 mV, respectively, it is likely that the reduction of current is induced by aggregated ABTS^•+^ molecule passing through the nanopore at different voltages. The values of current amplitude were fitted with a first-order polynomial function, which produces a slope of −0.706 and intercept of 49.262. However, based on the fitting curves, the values of dwell time are 54.5 ± 21.374 ms, 42.8 ± 20.181 ms, 10.3 ± 3.05 ms, 6.0 ± 1.744 ms, 4.0 ± 1.441 ms, at −400, −500, −600, −700, and −800 mV. Figure 3h shows an exponentially decaying function (*t*_*d*_ *~ e* ^*−v/v0*^) was employed to fit the dwell time dependent on the applied voltages. The histograms of the dwell time of translocation events were added to the supplementary material (see Additional file 1: Figure S4).

**Fig. 5:**
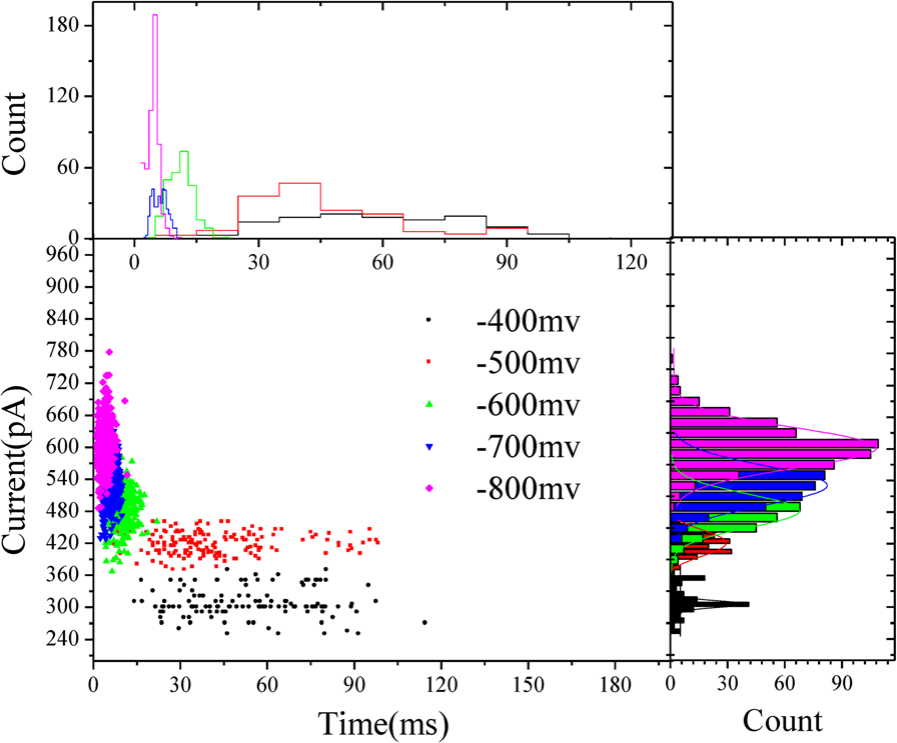
Two-dimensional scatter plots of current blockage versus events dwell time for each aggregated ABTS^•+^ at different voltages. Its corresponding histograms are put on the right and above. All histograms were fitted with Gaussian distribution

The current blockade versus events dwell time for each aggregated ABTS^•+^ at different voltages were fitted in two dimensional scatter plots (Fig. 5). All of the aggregated ABTS^•+^ show a cluster of events from −400 to −800 mV, the main event clusters are due to aggregated ABTS^•+^ passing through the HRPs modified solid-state nanopore.

In addition, the single translocation event at every voltage was analysed, and the current blockade was induced by the same size and charged substance. So, it is deemed that every translocation event was induced by the single aggregated ABTS^•+^. To analyse the translocation time of aggregated ABTS^•+^ in our experiments. The current blockade duration *t*_*d*_ is regarded as the dwell time of a single aggregated ABTS^•+^ from the location where it produced to the exit of nanopore. Here, another condition was considered, there may be some HRP enzymes immobilized on the entrance of nanopore, and it might catalyse the redox reaction. Therefore, other experiments were conducted to verify that the redox reaction occurred at the inner surface of a single nanopore rather than at the entrance. For the verification, unmodified by HRPs were applied and analysed. These nanopores were activated with 3-APTES. And the same concentration HRPs (10 ng/ml), ABTS (1.5 mM) and H_2_O_2_ (0.5 mM) were added to the trans compartment of nanopore, the negative biased voltage was applied in 0.1 M KCl, 0.1 M PBS, pH 7.0, due to the electrophoresis force, HRPs were unable to pass through the nanopore. Owing to the redox reaction, the aggregated ABTS^•+^ produced, but there were no translocation events found. It is possible that the aggregated ABTS^•+^ cause electrostatic effect with HRPs and prevent the aggregated ABTS^•+^ passing through the nanopore.

**Fig. 6:**
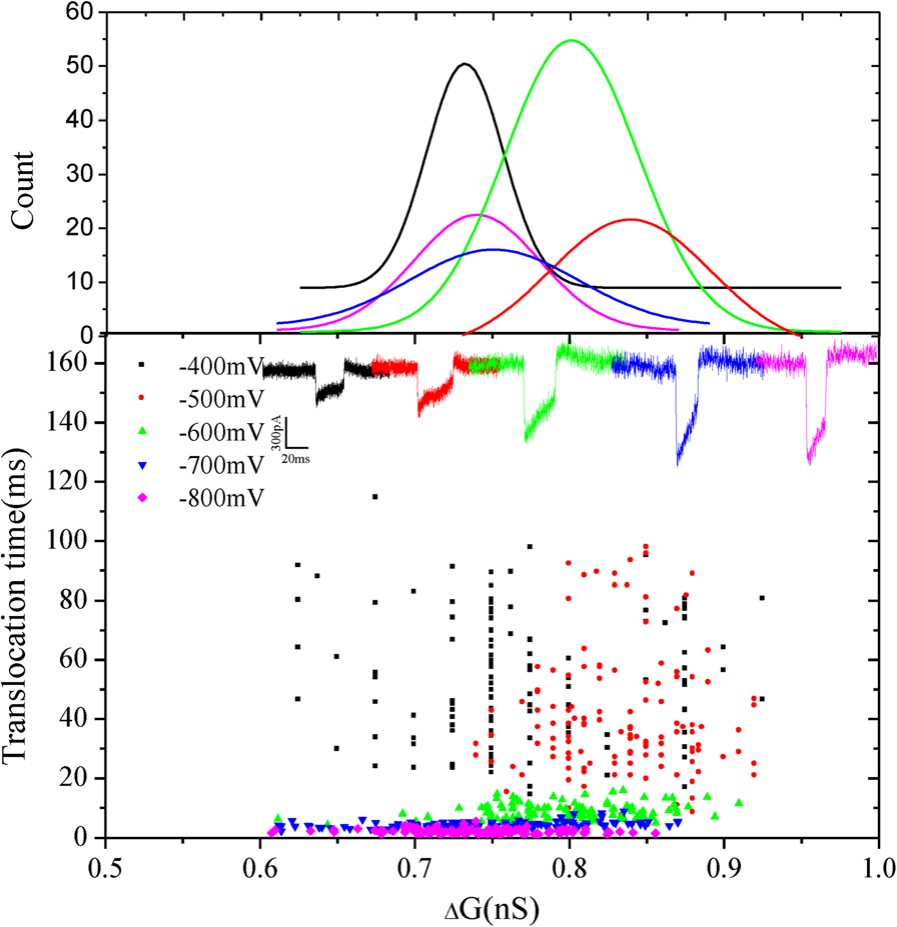
Schematic representation of two dimensional scatter plots of *ΔG* versus events dwell time for each aggregated ABTS^•+^ at different voltages. The corresponding fit curves were positioned above. The *insert* is the translocation events of different voltages (from −400 to −800 mV)

**Fig. 7:**
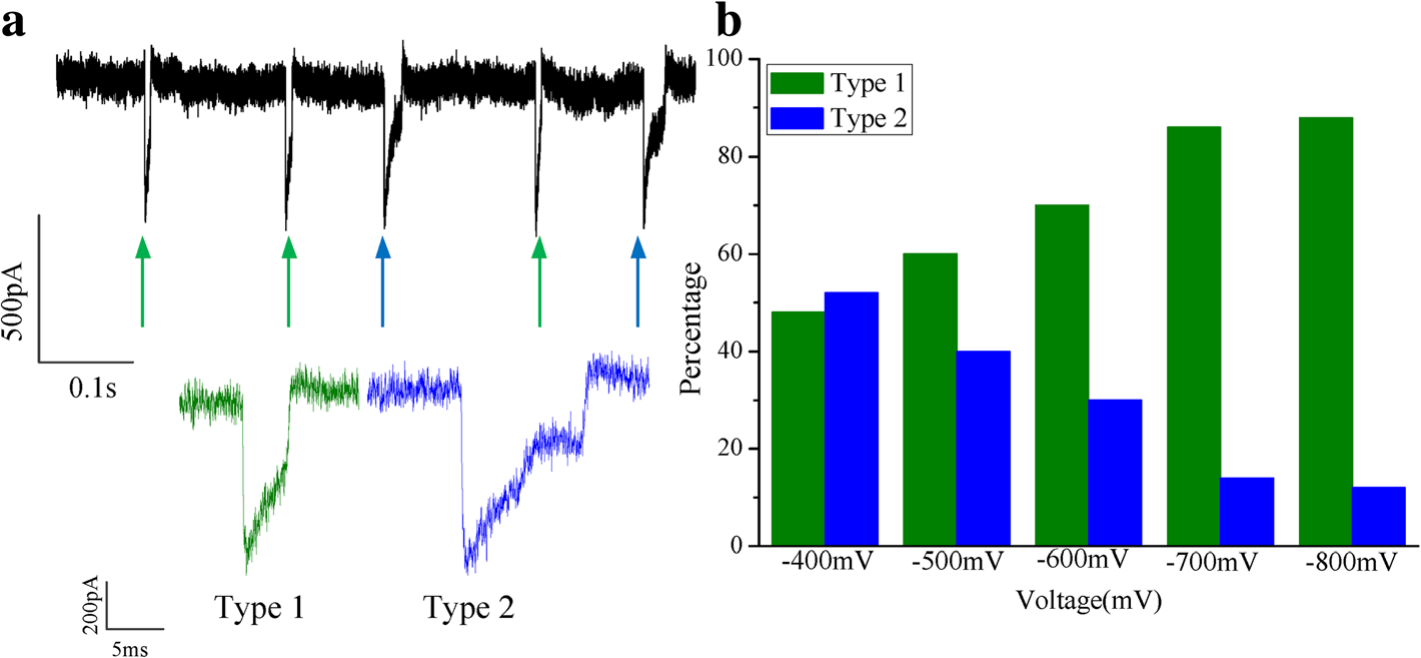
**a** Schematic of two type translocation events at −700 mV voltage. **b** The percentage of two types events at different voltages (−400, −500, −600, −700, −800 mV)

Figure 6 shows the two dimensional scatter plots of the change of conductance (*ΔG*) versus events dwell time for each aggregated ABTS^•+^ at different voltages. It can be found that change of conductance (*ΔG*) mainly concentrated in 0.8 nS. The shape of translocation events are almost the same. The mean value of *ΔG* is ~0.8 nS at different voltages. It can be speculated that the volume exclusion of every aggregated ABTS^•+^ molecule is almost same. It is possible that electrostatic and steric effects of aggregated ABTS^•+^ molecules may change the ionic current. After the analysis, two typical shapes of current traces with the positive charged aggregated ABTS^•+^ translocation were observed (Fig. 7). The translocation events at −700 mV as a representative. The percentage of two type events were analysed, and it can be observed that the percentage of type 1 events increased with the increase in voltage, on the other hand, the percentage of type 2 events was decreased. It was considered that higher voltage make the translocation faster than the lower voltage.

The current blockade signals revealed the size, conformation, and interaction of aggregated ABTS^•+^ passing through the single nanopore channel. For the change in current shape, the process of the changes were speculated. For the event 1, current signals have a typical fluctuation part with a deep intensity and a short dwell time. It is possible that the aggregated ABTS^•+^ passed through the nanopore from the place where it is produced. When the aggregated ABTS^•+^ passed through the nanopore, the ionic current of nanopore restored to the original level (Baseline) (*I*_*0*_). For the event 2, the current signals have a fluctuation part with a deep intensity and then have a horizontal stage. This shape of signals can be attributed to the electrostatic interaction of aggregated ABTS^•+^ with the HRPs at the exit of nanopore, and current was slowly recovered to the baseline. For better understanding of current change, we need to start with the open pore conductance change (*G*_*pore*_) at salt concentration (0.1 M KCl). As discussed in the previous studies, an equation of the open pore conductance of a negatively charged nanopore with a diameter of *d* and a length of *l* at low salt concentration can be described as

2 $$ {G}_{p ore}=\frac{\pi {d^2}_{p ore}}{4{l}_{p ore}}\left[\left({\mu}_{K^{+}}+{\mu}_{C{ l}^{-}}\right){n}_{K Cl}\cdot e+{\mu}_K\frac{4{\sigma}_p}{d_{p ore}}\right] $$

where *μ*_*K*_ and *μ*_*Cl*_ are the electrophoretic motilities of K^+^ and Cl^−^, *n*_*KCl*_ is the number density of K^+^ and Cl^−^, the elementary charge is *e, σ*_*p*_ is the surface charge density of the nanopore surfaces. In this experiment, the solid-state nanopore was chemically modified, and diameter of nanopore was changed. The surface charge density of the nanopore surface (*σ*_*p*_) cannot be obtained exactly. Therefore, the open pore conductance (*G*_*pore*_) was calculated based on equation (1). On account of equation (1), the open pore conductance (*G*_*pore*_) is ~7.5 nS. It is speculated that the change of conductance can be attributed to two reasons [15]. The first reason is that, the volume exclusion of ions in nanopore were occupied by the aggregated ABTS^•+^ molecules. As a result, the conductance of solid-state nanopore was decreased (*ΔG* ^*-*^). The second reason is that, some ions were brought from the nanopore by the aggregated ABTS^•+^ molecules which increased the conductance of solid-state nanopore. In these experiments, the ABTS^•+^ produced inside nanopore, and no ions were brought. Therefore, the change in the conductance of solid-state nanopore (*ΔG*) was only induced by the volume exclusion. So, the total change of the conductance can be described as

3 $$ \varDelta G=\varDelta {G}^{-} $$

The decrease in the conductance of solid-state nanopore is induced by the volume exclusion and it can be calculated by the following equation

4 $$ \varDelta {G}^{-}=\sigma \frac{\gamma \varLambda}{{\left( l+0.8 d\right)}^2} $$

where *γ* is particle shape factor which is the surface areas ratio of the same volume spherical and the particle. In this work, the aggregated ABTS^•+^ molecule was simplified to a global object, therefore the value of *γ* is 1 and *Λ* is the volume exclusion. The conductivity of bulk solution *σ* is 1.28 S/m, 0.1 M KCl (25 °C).

For the volume exclusion (*Λ*), we can deduce from the translocation events of some other molecules. For connecting the conductance change (*ΔG*) to the physical property of molecules, Ohm’s Law can be applied to the volume change of electrolyte solution based on the solid-state nanopore [59]. When a translocation event of a molecule in a cylindrical solid-state nanopore, the current decreased instantaneously. When the resistance of solid-state nanopore is the whole circuit resistance, the conductance change (*ΔG*) can be described by the following equation

5 $$ \varDelta G(t)=-{G}_{p ore}\frac{\varLambda (t)}{H_{eff}{A}_p}\left[1+ f\left({d}_m/{D}_p,{l}_m/{H}_{eff}\right)\right] $$

In this equation, *A*_*p*_ *H*_*eff*_ *= V*_*p*_ is the volume of solid-state nanopore, *f* (*d*_*m*_ */D*_*p*_ *, lm/H*_*eff*_) is correction factor (it ignored the surface charge effect), in our experiments, we simplified the aggregated ABTS^•+^ molecule to a global object; therefore, the correction factor is 1. The *d*_*m*_ */D*_*p*_ is the ratio of molecule diameter and nanopore diameter, The *lm/H*_*eff*_ is ratio of molecule effective length and naopore effective length. The expression (5) can be simplified as

6 $$ \varDelta G/{G}_{p ore}\approx \varLambda /{V}_p $$

The mean value of conductance (*G*_*pore*_) has been analysed of translocation events. From the equation (5), the mean value of volume exclusion (*Λ*) at different voltages (-400, -500, -600, -700, -800 mV) can be obtained. Meanwhile, the size of the used nanopore is known, the volume of nanopore (*V*_*p*_) is ~90746 nm^3^. On account of equation (4), the value of conductance change (*ΔG* ^*-*^) can be calculated as ~0.6 nS. The mean value of conductance change that obtained from the translocation events experiments at different voltages (−400, −500, −600, −700, −800 mV) is ~0.784 nS. It can be found that the calculated value is near to the experimental value.

In some previous investigation, hydrogen peroxide molecules have been achieved to be detected with different technologies. But, to detect hydrogen peroxide by nanochannel is rare. Tan et al. [3] differentiated disparate event signals when HRPs threaded into nanopore, there were ABTS and H_2_O_2_ in KCl solution. The different type signals with HRPs translocation were regarded as ABTS^•+^ passing through nanopore. Six typical events of the translocation of the product of enzyme catalysis substrates were analyzed. They speculated the probable process of every type. However, no enough evidences to testify. Mubarak Ali et al. have accomplished to detect the redox reaction products inner single conical nanochannels [58]. They found that the cationic radical ABTS^•+^ reduced the ion current in the HRP-nanochannel in a voltage-dependent fashion, consistent with voltage-dependent concentrations of ions in conical nanochannels. The magnitude of the current blockage was correlated with the H_2_O_2_ concentration in the solution.

## Conclusions

In conclusion, we fabricated a Si_3_N_4_ nanopore employing a FIB successfully, a single naonopore system whose surface was modified with covalently linked HRP enzymes. The effect of the immobilized HRPs enzymes in a single solid-state nanopore as a hydrogen peroxide (H_2_O_2_) sensor was affirmed by investigating products (ABTS^•+^) of the redox reactions occurring in presence of the substrates H_2_O_2_ and ABTS. The aggregated cationic radical ABTS^•+^ produced inside the solid-state nanopore and reduced the ionic current in the HRPs modified solid-state nanopore, are consistent with voltage-dependence. The current blockade trends showed linear dependence for applied biased voltages. The relationship between the dwell time versus applied biased voltage was the exponentially decaying (*t*_*d*_ *~ e* ^*−v/v0*^). Meanwhile, the aggregated ABTS^•+^ passed through the HRPs modified nanopores needed a −300 mV threshold voltage. The change of conductance (*ΔG)* has been calculated analytically and compared to the measured experimental values. The translocation events were produced by the certain size aggregated cationic radical ABTS^•+^. We expect that using solid-state nanopores will allow lowering the detection limit and improve the system sensitivity. For our solid-state nanopore system, the structure is simple; it is not susceptible to fouling and can be used multiple times.

## Additional file

Additional file 1: The instruments of our experiments used. **Figure S1.** The system include an Axopatch 700B (Molecular Devices, Inc., Sunnyvale, CA, USA). A double Faraday cage enclosure. The apparatus of our experiments used. **Figure S2.** The pictures of a custom-built Teflon cell with two Viton o-rings to separate the two side of chip. 1 Joint; 2 Teflon cell; 3 M5 plastic screw; 4 Viton o-rings. The experiments data of long duration translocation events of different voltages. **Figure S3.** The experiments data of long duration translocation events of different voltages from -400 to -800 mV in 0.1M KCl, 0.1 M PBS, pH 7.0. The histograms of the dwell time of translocation events. **Figure S4.** The histograms of the dwell time of translocation events. Based on the fitting curves, the values of dwell time are 54.5 ± 21.374 ms, 42.8 ± 20.181 ms, 10.3 ± 3.051 ms, 6.0 ± 1.744 ms, 4.0 ± 1.441 ms, at −400, −500, −600, −700, and −800 mV. The SEM images of nanopore silicon nitride thin film deposited on Si wafer. **Figure S5.** (a) The picture of experiments used Si_3_N_4_ nanopore. (b) The SEM image of nanopore silicon nitride thin film deposited on Si substrate. (c) (d) The SEM images of nanopore silicon nitride thin film and broken silicon nitride thin film. (e) The process of nanopore fabrication. (DOCX 2737 kb)

## Abbreviations

3-APTES: (3-Aminopropyl)triethoxysilane
ABTS: 3-ethylbenzothiazoline-6-sulfonic acid
EDC: N-(3-dimethylaminopropyl)-N’-ethylcarbodiimide
FIB: Focused ion beam
HRP: Horseradish peroxidase
KCl: Potassium chloride
NHS: N-hydroxysuccinimide
SEM: Scanning electron microscopy
SNR: Signal to noise ratio
