# Supplementary material for: Hydrogen Peroxide Sensing Based on Inner Surfaces Modification of Solid-State Nanopore
Source: Nanoscale Res Lett. 2017 Jun 20;12:422. doi: 10.1186/s11671-017-2190-x (PMC5478554; doi:10.1186/s11671-017-2190-x)
Supplement: Additional file 1: — The instruments of our experiments used. Figure S1. The system include an Axopatch 700B (Molecular Devices, Inc., Sunnyvale, CA, USA). A double Faraday cage enclosure. The apparatus of our experiments used. Figure S2. The pictures of a custom-built Teflon cell with two Viton o-rings to separate the two side of chip. 1 Joint; 2 Teflon cell; 3 M5 plastic screw; 4 Viton o-rings. The experiments data of long duration translocation events of different voltages. Figure S3. The experiments data of long duration translocation events of different voltages from -400 to -800 mV in 0.1M KCl, 0.1 M PBS, pH 7.0. The histograms of the dwell time of translocation events. Figure S4. The histograms of the dwell time of translocation events. Based on the fitting curves, the values of dwell time are 54.5 ± 21.374 ms, 42.8 ± 20.181 ms, 10.3 ± 3.051 ms, 6.0 ± 1.744 ms, 4.0 ± 1.441 ms, at −400, −500, −600, −700, and −800 mV. The SEM images of nanopore silicon nitride thin film deposited on Si wafer. Figure S5. (a) The picture of experiments used Si3N4 nanopore. (b) The SEM image of nanopore silicon nitride thin film deposited on Si substrate. (c) (d) The SEM images of nanopore silicon nitride thin film and broken silicon nitride thin film. (e) The process of nanopore fabrication. (DOCX 2737 kb) [file 11671_2017_2190_MOESM1_ESM.docx]

**Hydrogen Peroxide Sensing Based on Inner Surfaces Modification of Solid-State Nanopore**

LiBo Zhu, DeJian Gu and QuanJun Liu*

State Key Laboratory of Bioelectronics, School of Biological Science and Medical Engineering, Southeast University, No. 2, Sipailou, Nanjing 210096, People’s Republic of China

*S1 The instruments of our experiments used.*


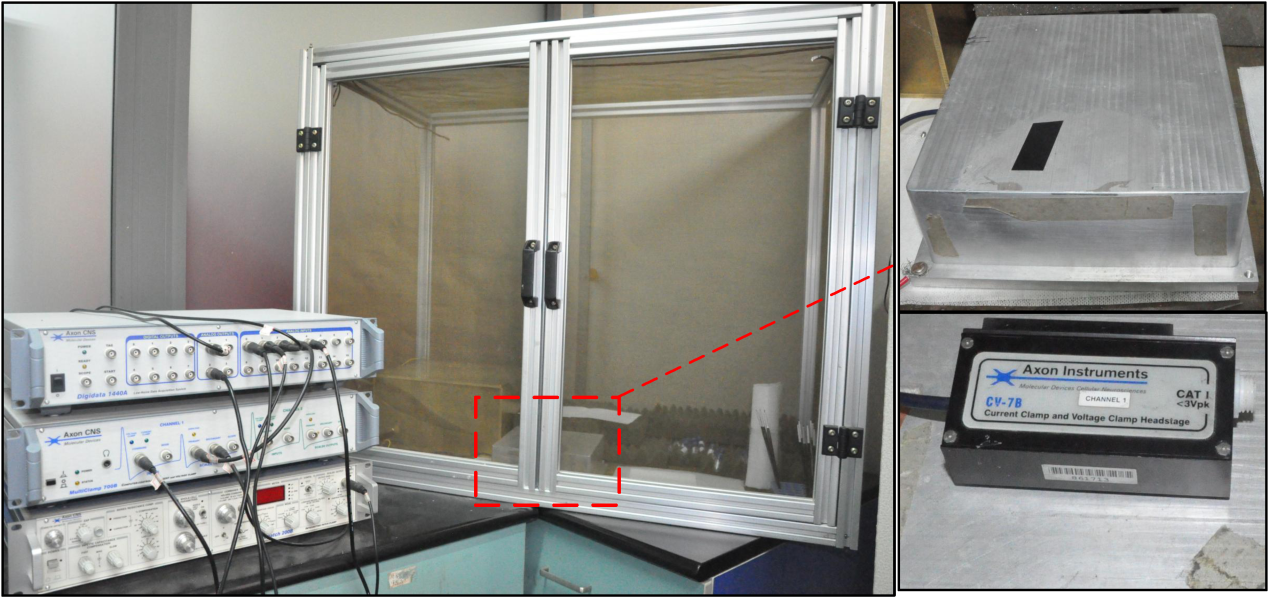


Figure S1. The system include an Axopatch 700B (Molecular Devices, Inc., Sunnyvale, CA, USA). A double Faraday cage enclosure.

*S2. The apparatus of our experiments used.*

*
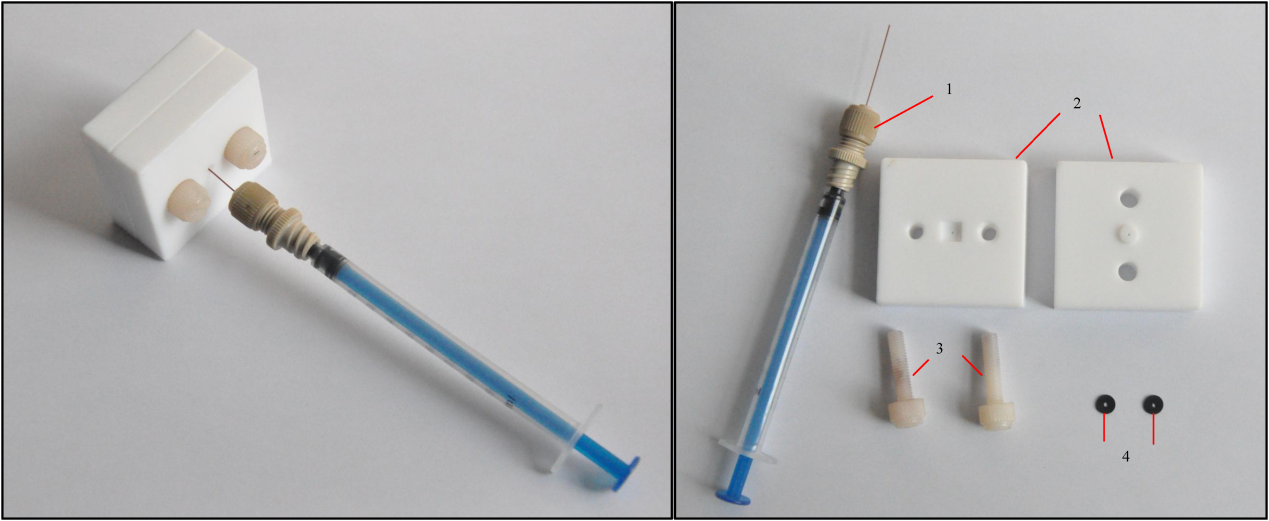
*

Figure S2. The pictures of a custom-built Teflon cell with two Viton o-rings to separate the two side of chip. 1 Joint; 2 Teflon cell; 3 M5 plastic screw; 4 Viton o-rings.

*S3 The experiments data of long duration translocation events of different voltages .*


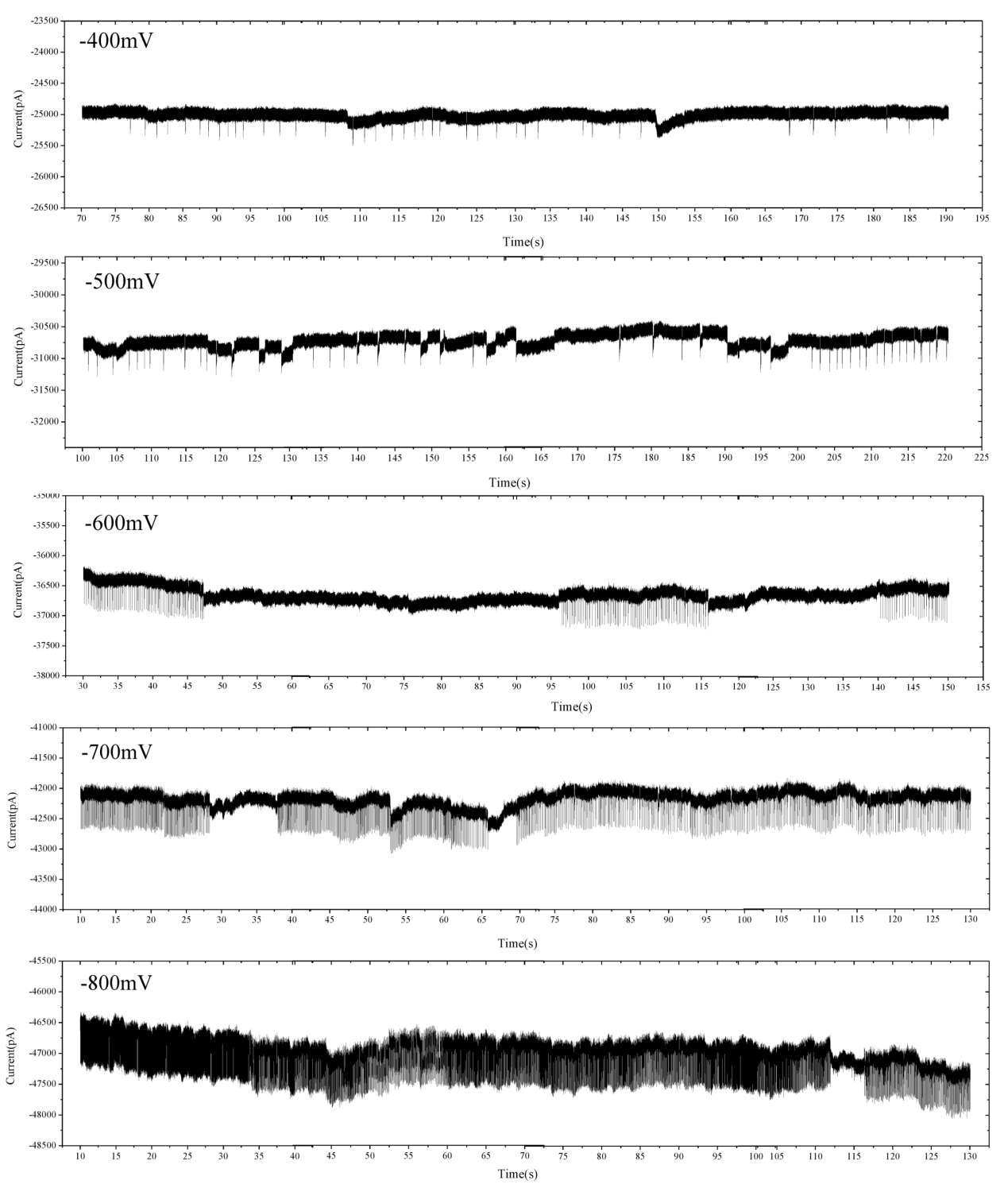


Figure S3. The experiments data of long duration translocation events of different voltages from -400 to -800 mV in 0.1M KCl, 0.1 M PBS, pH 7.0.


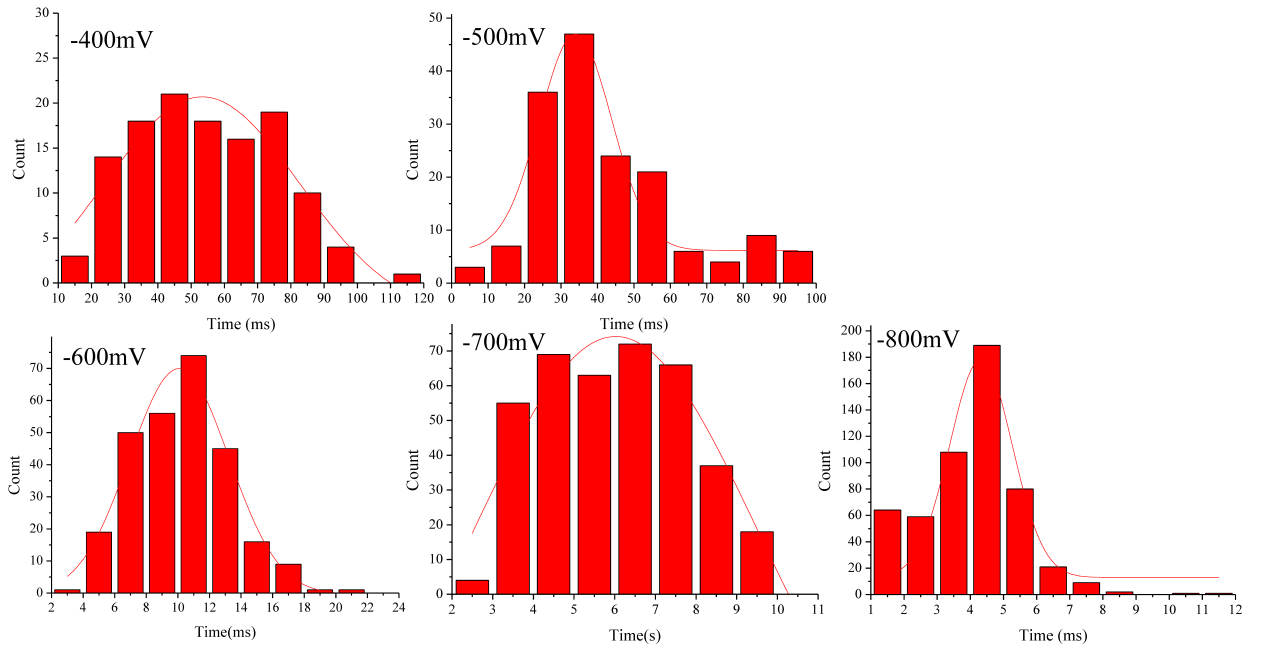


Figure S4. The histograms of the dwell time of translocation events. Based on the fitting curves, the values of dwell time are 54.5±21.374 ms, 42.8±20.181 ms, 10.3±3.051 ms, 6.0±1.744 ms, 4.0±1.441 ms, at -400, -500, -600, -700 and -800 mV.

*S5 The SEM images of nanopore silicon nitride thin film deposited on Si wafer.*


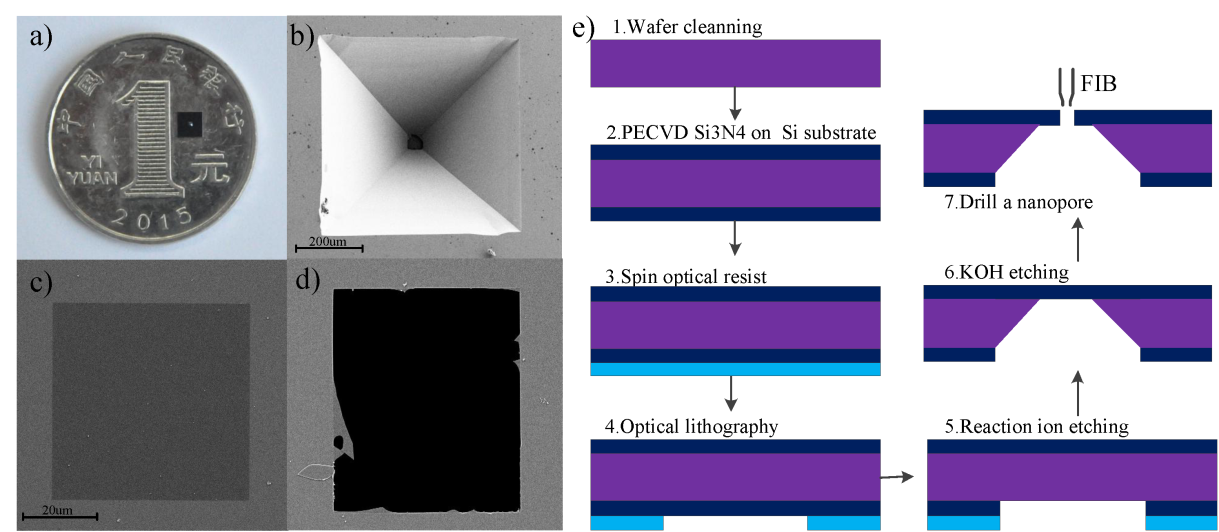


Figure S5. (a) The picture of experiments used Si_3_N_4_ nanopore. (b) The SEM image of nanopore silicon nitride thin film deposited on Si substrate. (c) (d) The SEM images of nanopore silicon nitride thin film and broken silicon nitride thin film. (e) The process of nanopore fabrication.
